# An ERP Study on Attraction Effects in Advanced L2 Learners

**DOI:** 10.3389/fpsyg.2021.616804

**Published:** 2021-09-27

**Authors:** Jing Bian, Hui Zhang, Chongfei Sun

**Affiliations:** ^1^School of Foreign Languages and Cultures, Nanjing Normal University, Nanjing, China; ^2^School of International Studies, Hangzhou Normal University, Hangzhou, China

**Keywords:** N400, P600, second language acquisition, agreement attraction, ERPs

## Abstract

In English, the rule of agreement is quite simple: verbs must agree with their subject head nouns in terms of number features. Despite this simplicity, agreement processing is always interrupted when the subject phrase of the sentence “The key to the *cabinets* is on the table,” contains two nouns with a mismatch in number features commonly known as attraction effects. This study used event-related potentials (ERPs) to examine whether late advanced second language (L2) learners can acquire native-like sensitivity of attraction effects. The results revealed that L2 learners showed ERP patterns qualitatively similar to native English speakers: ungrammatical verbs following singular attractors elicited a P600 effect relative to their grammatical counterparts, whereas this positivity was replaced by an N400 effect when plural attractors intervened between the subject head nouns and the verbs. Of particular interest, given that, compared to native speakers, the amplitude of the P600 effect elicited by L2 learners was smaller, there was a quantitative difference between native speakers and L2 learners. We proposed that these two ERP components represented the two processing routes of agreement: the P600 effect indexed a full, combinatorial process, which parsed morphosyntactic features between agreement controllers and targets, whereas the N400 effect indexed a shallow, heuristic process, which evaluated lexical associations between agreeing elements. Moreover, similar to native speakers, advanced L2 learners showed an asymmetrical pattern of attraction effects, in that plural attractors were interfered with ungrammaticality at disagreeing verbs, but they did not cause any difficulties in processing grammatical sentences at agreeing verbs. The overall results suggested that compared to native processing, L2 processing of complex agreement with attractor interference was shallower and therefore late advanced L2 learners could not achieve native-like attraction effects.

## Introduction

One of the major research questions of second language acquisition (SLA) concerns whether syntactic processing in late advanced second language (L2) learners may eventually become qualitatively similar to native language (L1) processing. Although a lot of evidence has proven the successful attainment of native-like processing by L2 learners in terms of simple or local grammatical phenomena such as number agreement (Frenck-Mestre et al., 2008; Dowens et al., 2010; Banon et al., 2014), word category (Weber-Fox and Neville, 1996; Rossi et al., 2006), and tense marking (Sabourin and Stowe, 2008; Weber and Lavric, 2008; Moreno et al., 2010), a few studies have been conducted to examine whether complex structural processing in L2 learners can be native-like. The shallow structure hypothesis (SSH; Clahsen and Felser, 2006a,b; Clahsen et al., 2010) is an SLA theory that makes a direct distinction between the simple grammatical rules and complex syntactic representations in L2 processing of syntax.

According to SSH, language processing can be divided into two different parsing routes: full parsing, which is responsible for constructing the hierarchical structure from syntactic information-based lexical entries; and shallow parsing, which is responsible for forming a less detailed representation based on the lexical-semantic information. The core claim of SSH is that L2 processing is shallower and less detailed than native processing and relies more on semantic or surface-level information when parsing abstract syntactic representations (Clahsen and Felser, 2006a). However, it does not mean that L2 learners are totally restricted to the shallow parsing route. In a newly published article on SSH, Clahsen and Felser (2018) clarified some misrepresentations and misunderstandings of this hypothesis. The first misinterpretation is that L2 speakers can never use syntactic representations in the computation of the sentence. Different from the traditional view of regarding grammatical knowledge as categorical property (“know” vs. “does not know”), Clahsen and Felser (2018) suggested that the differences between L1 and L2 grammar are gradient so that they can in principle be quantified. That is to say, the full parsing route or syntactic information is available to L2 learners in the computation of syntactic representations but it is weighted to be less in L2 processing compared to L1. Another misinterpretation of SSH is that shallow processing is specific to L2 learners. Based on the multiple pathways of language processing, SSH affirms that both full and shallow parsing routes operate in parallel, with none of them restricted to L1 and L2 processing. That is, even native speakers may process syntax in a shallow manner and L2 learners may process syntax in a deep manner. Moreover,SSH is also misinterpreted as a hypothesis stating that L2 processing can never become native-like. Although Clahsen and Felser (2006a) speculate that the limited use of grammatical knowledge may impede L2 parsing skills to determine whether or not L2 learners can develop a native-like processing of syntax depending on various factors such as the relative weighting of different information types, the exposure of L2 input, and proficiency.

As for the complex syntactic processing, Clahsen and Felser (2018) asserted that their original statement about SSH “during L2 processing, learners compute grammatical representations that lack a complex hierarchical structure (Clahsen and Felser, 2006b)” appearing to be too broad and general based on more recent empirical findings. Considering that native speakers may process complex strings or sentences in a shallow manner (e.g., Severens et al., 2008) and proficient L2 learners are sometimes found to apply the full parsing route during the processing of highly complex sentences (e.g., Felser and Drummer, 2017), the refined version of SSH asserts that what really distinguishes L2 processing from native processing is that L2 learners may prioritize semantic, pragmatic, or other types of non-grammatical information and underuse syntactic information during real-time processing (Clahsen and Felser, 2018). Alternatively speaking, the processing between L2 learners and native speakers may be qualitatively similar but quantitatively distinct. Specifically, on one perspective, they both are able to apply full and shallow parsing routes in the processing of complex syntax, on the other perspective, L2 learners display fewer weights of grammatical constraints, as well as more weights of non-grammatical constraints compared to native speakers. Because this study aims to investigate L2 processing of complex syntax, there is no doubt that hierarchically complicated structures, especially those including non-local dependencies, are most suitable for examining this question. For this reason, we chose the agreement attraction as the targeted structure in our investigation. This syntactic phenomenon includes the long structural distance between agreeing elements.

The remainder of the paper is structured as follows. We begin with a detailed explanation of agreement attraction and its theoretical hypotheses. Subsequently, we provide a review on previous event-related potential (ERP) studies concerning agreement attraction in L1 and L2 processing separately, focusing on the neurocognitive mechanisms underlying attraction effects for both L1 and L2 groups. After introducing the aims, methods, and results of the present investigation, we mainly focus on the discussion revolving around the ERP profiles of attraction effects to gain more insights into whether advanced L2 leaners can acquire the native-like processing of complex syntactic structures.

## Agreement Attraction

Linguistic elements intervening between the subject head noun and the verb always disrupt the processing of agreement and lead to syntactic violations, commonly known as agreement attraction. Previous studies on agreement attraction under the production paradigm have found that complex subject noun phrases (NPs) that contain two nouns with a mismatch in number features elicited an increased number of errors in verb number marking, yielding sentences like “^*^the key to the cabinets were lost,” where the verb erroneously agrees with the plural attractor immediately preceding it (e.g., cabinets) rather than the singular head noun of the subject NP (e.g., key) (Bock and Miller, 1991; Bock and Cutting, 1992; Vigliocco and Nicol, 1998; Eberhard, 1999). Moreover, the interference of plural attractors has also been observed in language comprehension experiments (Nicol et al., 1997; Pearlmutter et al., 1999; Wagers et al., 2009). For example, Nicol et al. (1997) found that reading times for grammatical verbs in case of the conditions of singular head nouns and plural attractors were significantly delayed relative to the conditions of both head nouns and attractors being singular, suggesting attraction effects in grammatical sentences. Pearlmutter et al. (1999) expanded on the previous research and observed that plural attractors embedded in prepositional phrase (PP) modifiers caused symmetrical interferences, affecting the processing of both grammatical and ungrammatical sentences. More recently, a series of self-paced reading studies conducted by Wagers et al. (2009) revealed an ungrammatical–grammatical asymmetry: attraction effects are limited to the agreement processing in ungrammatical sentences. As for the previously reported attraction effects in grammatical sentences, Wagers et al. (2009) argued that it might due to the spillover effect from the complexity of plural attractor nouns.

Attraction has been accounted for by a minimum of two theoretical hypotheses. One family of theories has proposed that attraction effects arise due to an inaccurate mental representation of the number features of the complex subject NP (Nicol et al., 1997; Vigliocco and Nicol, 1998; Franck et al., 2002; Eberhard et al., 2005; Staub, 2009, 2010). According to this view, attraction occurs when plural number features on attractors are spuriously transmitted throughout the structural representation of the subject NP, either through feature percolation (Bock and Cutting, 1992; Franck et al., 2002) or spreading activation (Hartsuiker et al., 2001; Eberhard et al., 2005). The mechanism of feature percolation posits that the number feature on the head noun has to be moved via unification to the sentential S-node to unify with the verb. However, if there is a PP modifier, the number feature of the attractor can also percolate up to the S-node, thus computing an incorrect agreement with the verb. In addition to the account of feature percolation, Eberhard et al. (2005) emphasized that the transmission of features could also be treated as an activation-like process, and that the number information in any position of the structural network can flow unobstructed to any other part of the structure, with the weights of the structural links in the hierarchical architecture modulating the strength of agreement between different constituents. The second type of theory suggests that attraction effects are generated from the cue-based working memory retrieval mechanism that is initiated for the purpose of checking verb agreement (Wagers et al., 2009; Dillon et al., 2013). The core idea behind this account lies on how the verb gets its agreement features. On the feature transmission account, the path of feature movement is driven forward from the subject to the verb. In contrast, on the retrieval account, the number feature of the verb is used as a cue to search backward for the subject in memory. If the number-matching head noun in the subject NP is correctly retrieved in the grammatical sentences, it is impossible for another mismatching noun to interfere with agreement computation. Thus, no disrupting effects due to agreement attraction will occur at the verb. In contrast, if the number feature of head noun is mismatched with the number feature of the verb, as in the ungrammatical context, the number-matching attractor might get the chance to be retrieved instead during the checking process, which causes the sentence processor not to notice the ungrammatical form of the verb. In summary, the difference between the feature transmission model and cue-based retrieval model lies on what causes the illicit number features to interfere with agreement computation. While the former holds that agreement attraction arises due to an unstable representation of the number feature of subject NP itself, the latter takes both the verb and subject NP into consideration and attribute attraction effects to the failure of constructing agreement dependencies between them.

## EPR Studies of Native Attraction Effects

Event-related potentials are the direct recordings of brain activities that are time-locked to sensory or cognitive events. With a high temporal resolution that can reach to milliseconds, ERP provides us with a useful tool for investigating the neurocognitive mechanisms underlying syntactic processing. Only a few studies have used ERPs to investigate attraction phenomena in L1 comprehension. Kaan (2002) examined the effects of interfering number properties on agreement processing by using Dutch subject-object-verb sentences. The results showed that ungrammatical verbs elicited a larger P600 effect in the conditions where both the subject and object were singular than in the conditions where the subject and object were both plural or mismatched in number features. Kaan (2002) suggested that agreement violations with plural features may be more complex to process compared to the violations only including singular nouns. Severens et al. (2008) investigated attraction interferences on the comprehension of Dutch agreement. They found a P600 effect elicited by disagreeing verbs when complex subject NPs contained plural attractors with mismatching number features (i.e., singular head/plural attractor), and an N400 effect elicited by disagreeing verbs when complex subject NPs contained singular attractors with matching number features (i.e., singular head/singular attractor). The two ERPs components, as suggested by the authors, represented the two parsing routes of agreement processing: the N400 effect indexed a shallow analysis of simple match conditions of agreement violation, and the P600 effect indexed a deeper syntactic analysis of more complex mismatch conditions of agreement violation. Shen et al. (2013) explored interfering effects of attractors on subject-verb agreement processing in an auditory experiment. It is observed that agreement violations without intervening attractors elicited the anterior negativity and the later posterior positivity (P600). However, no difference on the EPR pattern was found between grammatical and ungrammatical verbs when a singular subject noun was followed by a plural attractor. These results showed that plural attractors created an “illusion of grammaticality” (Phillips et al., 2011), making it no more difficult than grammatical agreement to process. Tanner et al. (2014) recorded ERPs in response to attraction effects under both grammatical and ungrammatical conditions. The results showed that disagreeing verbs in the singular attractor condition (singular head/singular attractor) elicited early positivity (P2) and late positivity (P600), but disagreeing verbs in the plural attractor condition (singular head/plural attractor) only showed a small P600 effect. The authors interpreted the reduction of P600 effect as evidence that the plural attractor could intervene in the processing of agreement. Santesteban et al. (2017) investigated attraction effects in antecedent-clitic dependencies in Spanish. It is found that the violations of pronominal dependencies elicited the biphasic frontal negativity P600 pattern when sentences contained a matching singular attractor, but a P600 when they contained a mismatching plural attractor. They argued that the absence of frontal negativities in ungrammatical sentences with plural attractors might be due to the possibility that the plural attractor NPs were misidentified as the plural clitic antecedent NPs.

In summary, ERP findings on attraction effects in native processing were mixed. Previous studies observed that attraction effects led to the reduction of P600 effects (Kaan, 2002; Tanner et al., 2014, 2017), the absence of diverse negative components, such as the posterior early negativity (Shen et al., 2013) and the frontal negativity (Santesteban et al., 2017), as well as the modulation of ERP components in different attractor conditions [the N400 effect in the singular attractor condition vs. the P600 effect in the plural attractor condition (Severens et al., 2008)].

## L2 Studies of Agreement Attraction

From the perspective of L2 acquisition, the structure of agreement attraction has always been applied to test the sensitivity of L2 learners to morphological processing (Jiang, 2004; Chen et al., 2007; Tanner et al., 2012; Jegerski, 2016). In Jiang (2004), for example, both proficient native Chinese speakers of English as a second language (ESL) and English native speakers were asked to react to the following two conditions of materials in a self-paced reading experiment.


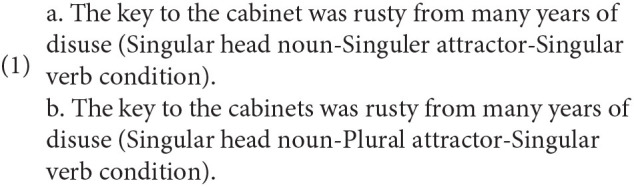


Reading times were measured to determine whether the morphological knowledge of agreement was a part of the integrated linguistic competence of participants. The author found that, whereas native speakers took significantly longer reading times to process SPS sentences at the critical verb, there is no significant difference in reading times between SPS and SSS conditions for L2 learners. These results indicated that non-native speakers did not show attraction effects. Jiang (2004) suggested that the lack of sensitivity for non-native subjects to verbal number morphology might have something to do with the fact that grammatical number is not inflected in their L1, Chinese.

More recently, another self-paced reading experiment by Jegerski (2016) also tested the attraction effect of L2 agreement processing. This investigation used an attraction paradigm to examine the online comprehension of Spanish subject-verb agreement by the three groups of participants: native speakers, near-native, and advanced learners of Spanish. Experimental stimuli are illustrated in (2a) and (2b).


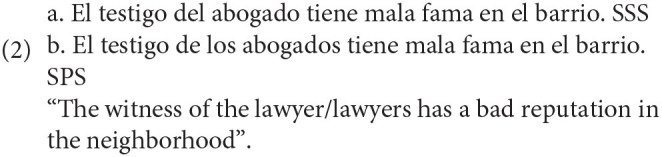


For the stimuli in (2), the region of interest included the verb region (tiene), the post-verbal region (mala fama), and the final region (en el barrio). It was observed that attraction effects were found in both L1 group and near-native L2 group. The advanced L2 participants, in contrast, did not show any effects or spill effects of attraction. The results of this experiment confirmed that non-native speakers were able to exhibit attraction effects while processing verbal number in Spanish.

In addition to the abovementioned self-paced reading research, ERP technology has also been used to investigate attraction effects in L2 agreement processing. For example, Chen et al. (2007) included an attraction manipulation in a study on the morphosyntactic processing of subject-verb agreement. The ERP results showed that, while the subject-verb violation elicited a typical biphasic pattern of LAN-P600 for native speakers, it invoked an unexpected negativity around 600 ms for L2 learners. Furthermore, of particular relevance to the present study was the result that L2 learners exhibited an N400-P600 pattern when processing grammatical verbs following plural attractors (singular head/plural attractor), in comparison with grammatical verbs following singular attractors, whereas the comparison between these two grammatical conditions elicited no effects for native participants. These findings demonstrated that although L2 learners were not sensitive to the global agreement between the head noun and verb, they showed a strong sensitivity to the local relation between the attractor and verb.

Tanner et al. (2012) used ERPs to examine whether L1 Spanish–L2 English participants were sensitive to agreement errors by manipulating plural or singular attractors intervened between the head noun and the verb (e.g., The winner of the big trophies/trophy has/have...). The results showed that subject-verb violation elicited a P600 effect in both late proficient Spanish–English bilinguals and native English speakers, with the size of P600 effect being smaller in the bilingual group. Moreover, in both groups, the P600 effect was smaller for ungrammatical verbs following plural attractors, suggesting attraction effects. According to the results, Tanner et al. (2012) concluded that even late L2 learners can exhibit a processing qualitatively similar to native speakers although there may be quantitative differences between them.

In summary, previous ERP and self-paced reading experiments have yielded mixed findings regarding attraction effects on L2 studies. On one hand, highly proficient L2 learners exhibited similar sensitivity to attraction effects with native speakers when their L1 and L2 shared the similar systems with respect to agreement realization (Tanner et al., 2012; Jegerski, 2016). On the other hand, the findings of L2 sensitivity to attraction are more controversial when the corresponding grammatical features are absent in the L1 of L2 learners. For example, Jiang (2004) found no attraction effects in native Chinese speakers, but Chen et al. (2007) reported that Chinese L2 learners of English had a strong online sensitivity to attraction. The divergence of the results may be explained by the different experimental methods and inconsistent levels of proficiency of L2 participants. Moreover, both Jiang (2004) and Chen et al. (2007) analyzed attraction effects within grammatical conditions, so it is unclear whether their results can be generalized to ungrammatical conditions. Given that grammatical–ungrammatical asymmetry is a crucially important characteristic of attraction (Wagers et al., 2009; Tanner et al., 2014), limiting the investigation to grammatical sentences cannot provide a comprehensive analysis of L2 attraction effects. For this reason, this study aims to test whether highly proficient Chinese learners are able to show native-like sensitivity to attraction effects when tested with both grammatical and ungrammatical conditions.

## The Present Study

The present study aimed to use ERPs to investigate attraction effects in proficient Chinese L2 learners. We focused on the interference effects from plural attractor nouns in complex subject NPs as singular attractor nouns have been reported to exhibit little interference in comprehension (Pearlmutter et al., 1999; Thornton and MacDonald, 2003). Of particular interest, this study was to determine whether highly proficient L2 learners could exhibit native-like sensitivity to attraction effects. Under the revised version of SSH, one of the possibilities is that the processing of complex syntax in late advanced L2 learners is predicted to be qualitatively similar to and quantitatively different from native speakers, with reduced weights of syntactic parsing route and/or increased the strength of the shallow parsing route applied in L2 syntactic processing relative to L1. If this is true, the ERP profile of attraction interferences should be similar between the native and L2 processing of agreement, but the amplitude of ERP component reflective of morphosyntactic processing (P600 and, possibly, LAN) may be smaller and/or the amplitude of ERP component reflective of semantic processing (N400) may be larger for L2 learners than native speakers.

It should be noted that, in the traditional SLA studies, the elicitation of N400 in response to morphosyntactic violations has always been regarded as evidence to prove that syntactic processing of L2 learners is qualitatively different from that of native speakers (e.g., Weber and Lavric, 2008; McLaughlin et al., 2010). However, this inference is premised on the assumption that no native speakers show “non-native-like” ERP components (Tanner, 2019). Recently, a few studies on language processing have suggested that there are robust individual differences in the type of ERP responses to agreement violations among both L2 learners and native speakers, with some exhibiting N400 effects and others exhibiting P600 effects (Tanner et al., 2013, Tanner and van Hell, 2014, Faretta-Stutenberg and Morgan-Short, 2018). Considering that the systematic variability in the effects of N400 and P600 appears to be a general phenomenon of language comprehension, this study also attempted to compare the individual variation in ERP profiles between native speakers and L2 learners so as to provide more solid evidence to L2 processing of complex syntax.

## Methods

### Participants

22 (12 women) native Chinese speakers who learned ESL participated in this experiment. They were graduate students majoring in English (mean 27 years, range 25–28) from Nanjing Normal University and Nanjing University. They have been learning English for more than 13 years (mean 15, range 13–17) with no exposure to English and other languages before puberty (age of acquisition: 11–13). All the native Chinese participants had also studied other L2s, including German (*n* = 5), French (*n* = 10), Russian (*n* = 1), and Japanese (*n* = 6). To make sure of the upper limits of L2 grammatical processing ability, only L2 participants who scored excellence (higher than 80 out of 100) on the test for English majors (TEM) level eight (the highest level) were recruited for the current investigation. L2 participants were required to rate their English proficiency on a one to seven Likert scale, and the mean scores were 5.59 for reading (SD = 0.50), 5.23 for speaking (SD = 0.75), 5.05 for listening (SD = 0.79), and 4.86 for writing (SD = 0.89). Two participants were excluded due to an excessive amount of blink artifacts in the raw electroencephalogram (EEG). Therefore, the data of 20 L2 learners (12 women) were used in the final analysis.

20 native speakers of English (11 women) (mean 24, range 23–25) were recruited as the control group, against which L2 learners could be compared. The native speakers were the exchange students from Australia, New Zealand, UK, and USA who were learning Chinese at Nanjing Normal University and Nanjing University. One participant was excluded due to an excessive amount of blink artifacts in the raw EEG. Thus, 19 English native speakers were included in the final analysis. All participants were right-handed and had normal or corrected-to-normal vision. They were paid for their participation in the experiment.

### Materials

120 quadruplets were constructed as experimental materials in a 2 × 2 design with grammaticality (grammatical and ungrammatical) and attractor number (singular and plural) as the factors, resulting in four sentence types (see Table 1). Each sentence contained a preamble where a subject head noun was followed by a PP modifier.

**Table 1:** Example experimental sentences.

**Grammaticality**	**Attractor number**	**Example sentence**
Grammatical	Singular attractor	The writer of the script was very popular
Ungrammatical	Singular attractor	The writer of the script were very popular
Grammatical	Plural attractor	The writer of the scripts was very popular
Ungrammatical	Plural attractor	The writer of the scripts were very popular

The grammaticality of the sentence was manipulated by varying the number of the critical verb (was or were), and the number of attractor nouns embedded in the PP was also manipulated as plural or singular. Some of the sentence preambles were selected or adapted from the published materials investigating attraction effects (Bock and Miller, 1991; Chen et al., 2007; Wagers et al., 2009; Tanner et al., 2014), others were newly constructed. The four conditions of each sentence were distributed across four separate experimental lists in a Latin Square design such that each participant was presented with only one condition of each sentence and there were an equal number (30) of critical trials in each condition per list. Additional set of 120 filler sentences were also selected. Half of the filler sentences were ungrammatical and contained different types of grammatical violation (including errors in the past tense, word order, and simple subject-verb agreement). Thus, each list thus consisted of 240 sentences, half of which were ungrammatical. Each list was pseudorandomized to guarantee no more than three trials from any single condition and no more than three grammatical or ungrammatical trials occurred in succession. To ensure that any effects of attractor number were due to the differences in number marking rather than plausibility differences, another group of 25 Nanjing Normal University graduates majoring in English were asked to rate the plausibility of the sentence preambles [e.g., *the key to the cabinet(s)*] on a scale of 1 (implausible) to 5 (plausible). They were explicitly instructed to judge the “plausibility” and were given some clearly plausible and implausible examples.

Mean scores on the plausibility were 4.31 (SD = 0.51) for singular head noun, singular attractor preambles, and 4.20 (SD = 0.53) for singular head noun, plural attractor preambles. The paired-sample *t*-test showed that these two types of sentence preambles did not differ significantly [*t*_(24)_ = 0.703, *p* > 0.1).

### Procedure

Participants were tested in a single session lasting approximately 90 min (including about 25 min of experimental preparation). Each session began with 10 practice trials, and the practice sentences were similar to the test sentences but were not used in the formal experiment. Each participant was randomly assigned to one of the stimulus lists and seated in quite an experimental room in front of a display screen. Each sentence was presented visually word-by-word in the center of the screen. The presentation was as follows: a fixation mark was displayed for 500 ms followed by a blank screen for 1,000 ms, followed by a stimulus sentence. Each word of the sentence was presented on the screen for 500 ms, followed by a 200-ms blank screen between words. After the end of each sentence, there was a 500-ms blank screen, followed by question prompts for the grammatical judgment, the word “yes” for grammatical sentences and the word “no” for ungrammatical sentences. If participants gave no response in 2,000 ms, the question prompts would disappear. The hand assignment of response type was counterbalanced across participants. When reading the sentences, participants were instructed to minimize movements and did the grammaticality judgment as accurately as they can.

### EEG Recording and Analysis

Electroencephalogram activity was recorded from 32-channel Ag/AgCI electrodes mounted on an elastic cap (Electro-cap International, Eaton, OH, USA) according to the extended 10–20 systems. All electrodes were referenced online to the left mastoid and offline re-referenced to the linked left and right mastoids. To control for eye-movement artifacts, the electrooculograms (EOGs) were recorded by the two electrodes, one placed above the left eye and one placed at the outer canthus of the right eye. Electrode impedance was kept at a value <5 kΩ. EEG was amplified by an amplifier with a band pass of 0.05–100 Hz and was digitized at a sampling rate of 1,000 Hz.

Epochs ranged from pre-stimulus 200 ms to post-stimulus 1,000 ms relative to the critical verbs were cut out from the continuous EEG data. Baseline correction was conducted in reference to the 200 ms pre-stimulus interval. The epochs were averaged at each electrode site in each condition for each participant. A band pass filter of 0.016–30 Hz was applied for the grand average waveforms over participants. Trials with muscular and ocular artifacts were excluded from the analysis.

Time windows were selected based on previous studies, together with the components of interest and visual inspection of the waveforms: 300–500 ms for the negativities (LAN or N400) and 500–800 ms for a late positivity (P600). To investigate the topographic difference, the data from midline (Fz, Cz, and Pz), medial (right hemisphere: F4, C4, and P4; left hemisphere: F3, C3, and P3), and lateral (right hemisphere: F8, T4, and P6; left hemisphere: F7, T3, and P5) were selected separately. A repeated measures ANOVA was conducted by means of grammaticality (grammatical and ungrammatical) and attractor number (singular and plural) as a within-participant factors, and group (native speaker and L2 learners) as a between-participant factor. ANOVAs also included laterality (left, midline, and right) and anterior/posterior (anterior, central, and posterior) additional within-subject factors. The Greenhouse–Geisser correction was applied to repeated measures with >1 degree of freedom in the numerator. A Bonferroni correction was applied for follow-up tests to control for Type I error. All values of *p* are reported after applying the Bonferroni correction. Effect sizes were reported using partial eta-squared (η^2^) for ANOVAs and Cohen's *d* for *t*-tests (Cohen, 1988; Lakens, 2013).

It is noted that, as the present study mainly focuses on interference effects of plural attractors on the native and L2 processing of agreement, we only report grammaticality effect and interactions with grammaticality to streamline the ERP analysis to the motivated experimental comparisons.

## Results

### Behavioral Results

The main proportion of sentences judged correctly across four conditions were presented in Table 2 (L2 learners) and Table 3 (native speakers). Repeated measures ANOVA on arcsine-transformed proportions judged correctly showed a main effect of grammaticality [*F*_(1,37)_ = 17.479, *p* = 0.000, η^2^ = 0.321], driven by the fact that both L2 learners and native speakers were more accurate in judging grammatical sentences than ungrammatical sentences. However, the interaction between grammaticality and the attractor number did not reach the significance [*F*_(1,37)_ = 2.563, *p* = 0.118, η^2^ = 0.065]. Importantly, no main effect of the group or the interactions relevant to the group were found, indicating that both groups showed similar profiles of attraction interference.

**Table 2:** Means and stand errors for behavioral judgment by L2 learners.

	**Grammatical [95% IC]**	**Ungrammatical [95% IC]**
Singular attractor	0.93 (0.08) [0.90, 0.96]	0.85 (0.16) [0.80, 0.91]
Plural attractor	0.91 (0.10) [0.87, 0.95]	0.79 (0.18) [0.74, 0.87]

**Table 3:** Means and stand errors for behavioral judgment by native speakers.

	**Grammatical [95% IC]**	**Ungrammatical [95% IC]**
Singular attractor	0.91(0.06) [0.88, 0.94]	0.88(0.08) [0.83, 0.92]
Plural attractor	0.90(0.07) [0.87, 0.94]	0.81(0.12) [0.76, 0.87]

### ERP Results

**Figure 1:**
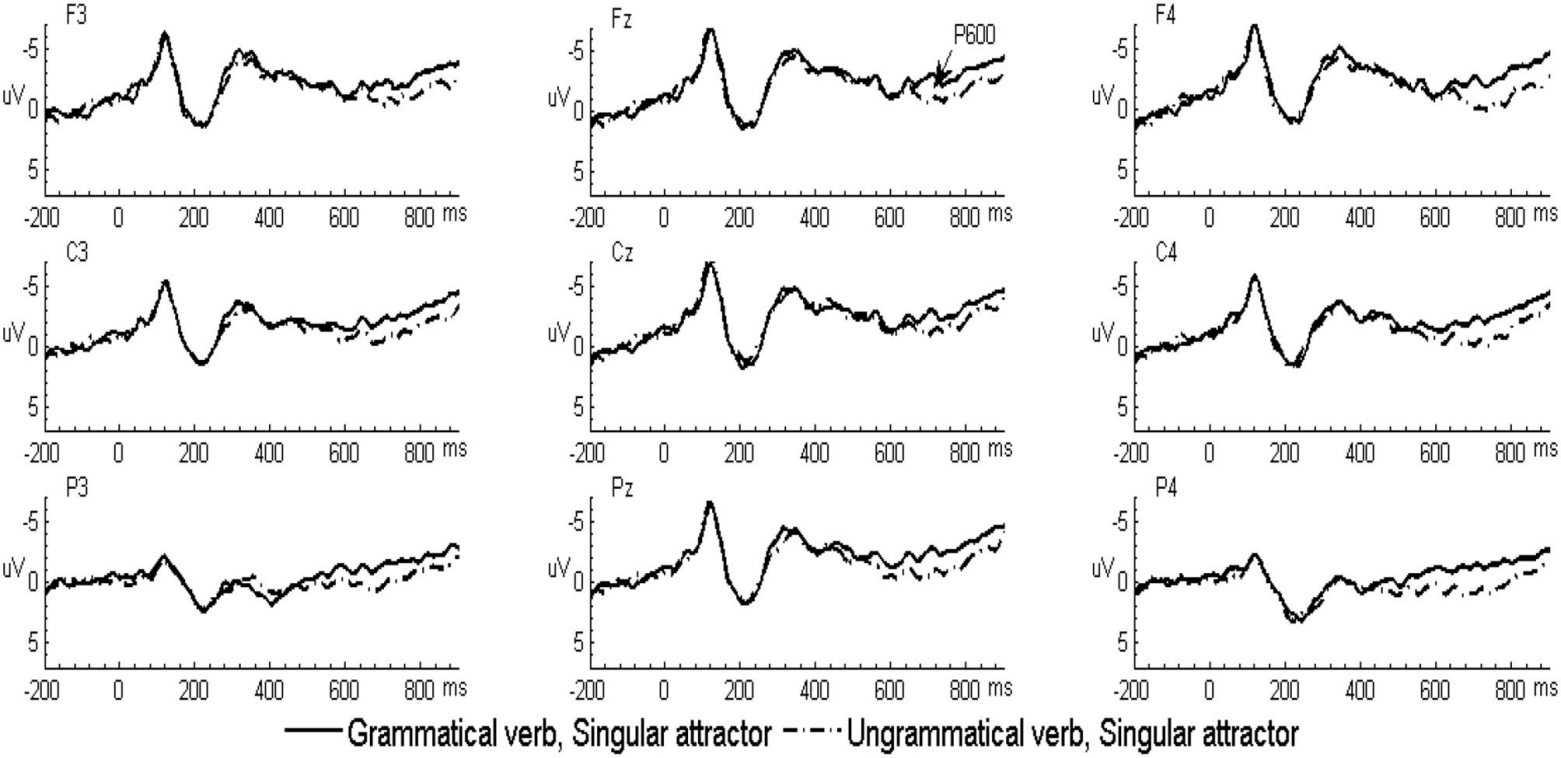
Grand-averaged event-related potential (ERP) waveforms for the grammatical (solid line) and ungrammatical (dashed line) verbs following singular attractors in the second language (L2) learner group.

**Figure 2:**
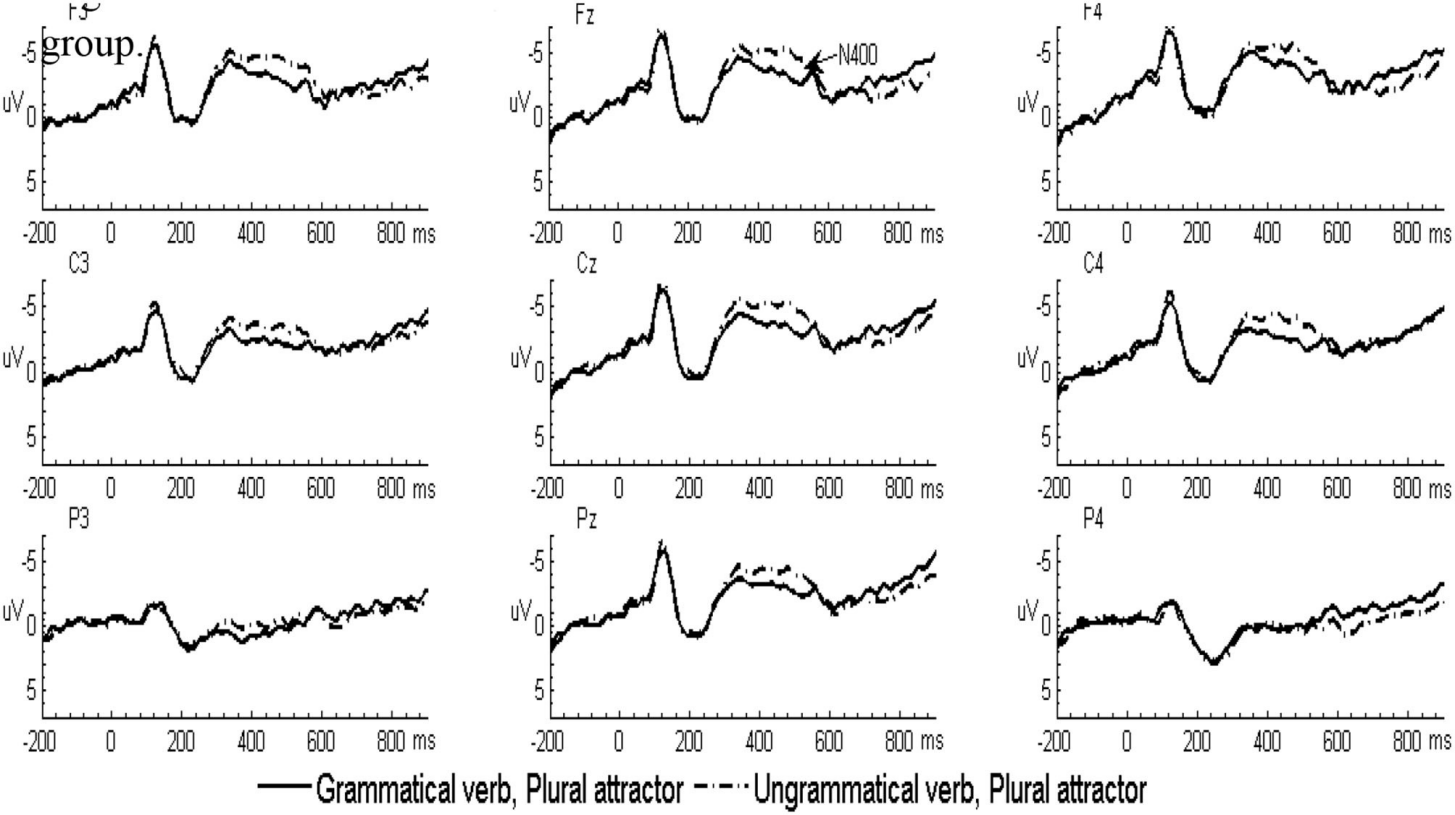
Grand-averaged ERP waveforms for the grammatical (solid line) and ungrammatical (dashed line) verbs following plural attractors in the L2 learner group.

**Figure 3:**
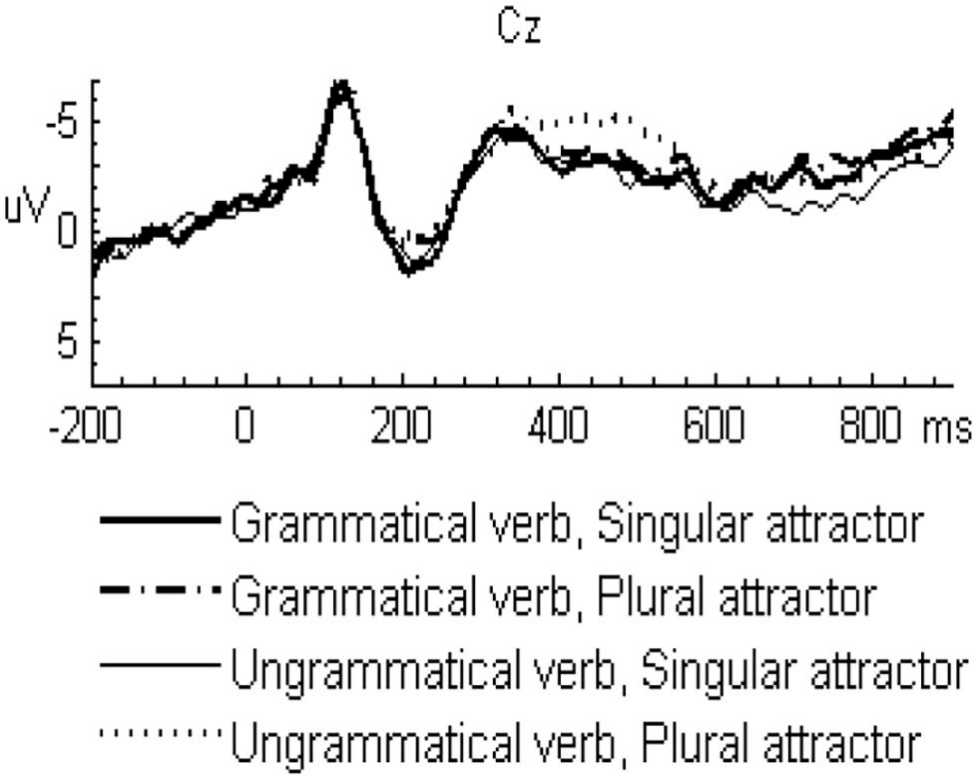
Grand average ERP waveforms for all four experimental conditions in the L2 learner group at midline vertex electrode Cz.

**Figure 4:**
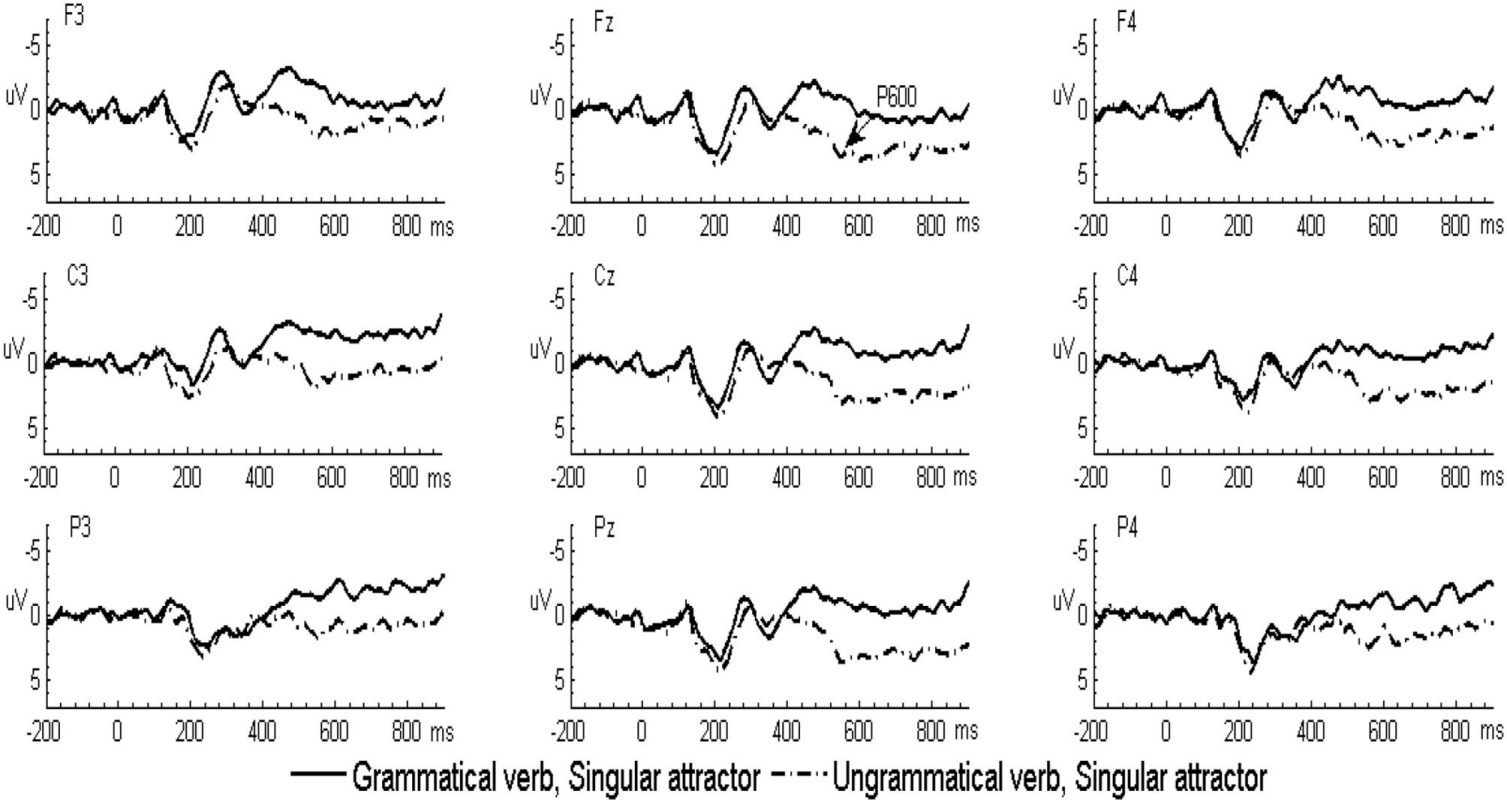
Grand-averaged ERP waveforms for the grammatical (solid line) and ungrammatical (dashed line) verbs following singular attractors in the native speaker group.

**Figure 5:**
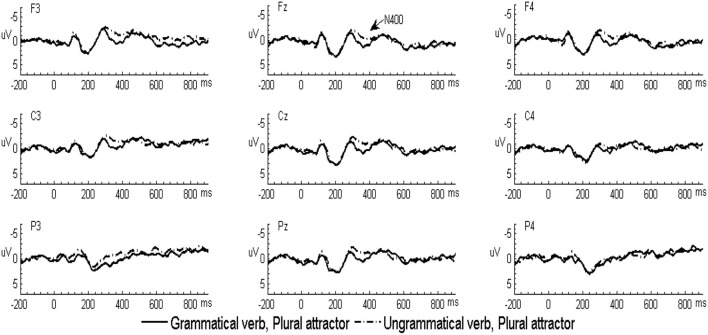
Grand-averaged ERP waveforms for the grammatical (solid line) and ungrammatical (dashed line) verbs following plural attractors in the native speaker group.

**Figure 6:**
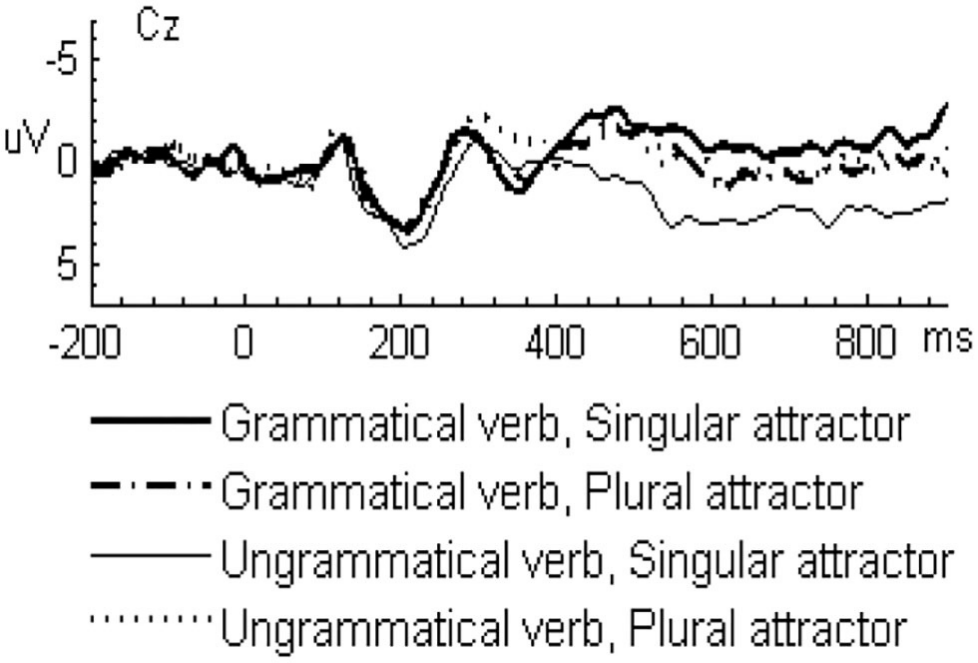
Grand-average ERP waveforms for all four experimental conditions in the native group at midline vertex electrode Cz.

In the L2 learners group, the grand-averaged ERP waveforms comparing grammatical and ungrammatical verbs in the singular attractor condition were displayed in Figure 1 and for the plural attractor condition in Figure 2. Waveforms for all four conditions were displayed in Figure 3. The grand-averaged ERP waveforms comparing the same conditions in the native group were displayed in Figures 4–6. Visual inspection of the waveforms showed the similar ERP patterns between native speakers and L2 learners: ungrammatical verbs in singular attractor conditions elicited late positivity around 500 ms, as compared to their grammatical counterparts, whereas in plural attractor conditions, this positivity became absent and the negativity was invoked instead by ungrammatical verbs around 300 ms. The results of the omnibus ANOVA are provided in Table 4.

**Table 4:** Results of the omnibus ANOVAs across native speakers and L2 learners.

	**300–500 ms**			**600–80 ms**		
	***F***	***p***	***η^2^***	***F***	***p***	***η^2^***
G	0.433	0.515	0.012	9.540	0.004^**^	0.205
G^*^Group	0.641	0.428	0.017	1.297	0.262	0.034
A^*^G^*^Group	0.155	0.696	0.004	0.045	0.833	0.001
G^*^A	11.502	0.002^**^	0.237	13.312	0.001^**^	0.265
G^*^A^*^Group	0.280	0.600	0.008	3.146	0.084^+^	0.078
G^*^L	0.452	0.634	0.012	2.260	0.114	0.058
G^*^L^*^Group	0.356	0.697	0.010	0.717	0.486	0.019
G^*^A^*^H	3.514	0.037^*^	0.087	0.745	0.458	0.020
G^*^A^*^L^*^Group	0.756	0.469	0.020	0.573	0.538	0.015
G^*^AP	0.004	0.983	0.000	4.034	0.026^*^	0.098
G^*^AP^*^Group	0.569	0.513	0.015	0.475	0.606	0.013
G^*^A^*^AP	2.324	0.127	0.059	0.468	0.550	0.012
G^*^A^*^AP^*^Group	0.414	0.575	0.011	1.141	0.336	0.030
G^*^L^*^AP	2.585	0.045^*^	0.065	0.284	0.843	0.008
G^*^L^*^AP^*^Group	0.428	0.769	0.011	1.141	0.336	0.030
A^*^L^*^AP^*^Group	1.800	0.144	0.046	1.958	0.120	0.050
G^*^A^*^L^*^AP	0.268	0.851	0.007	0.765	0.513	0.020
G^*^A^*^L^*^AP^*^Group	0.520	0.672	0.014	0.820	0.482	0.022

*G, Grammaticality; A, Attractor number; AP, Anterior/Posterior; L, Laterality*.

*0.05 ≤ +p ≤ 0.1*,

* *p ≤ 0.05*,

** *p ≤ 0.01*.

#### 300–500 ms Time Window

As shown in the table, statistical analyses revealed a significant grammaticality by attractor number interaction. Follow-up analyses combing both natives and L2 learners revealed that this interaction was driven by larger negativity to ungrammatical verbs in the plural attractor condition (*p* = 0.003, *d* = 0.58). However, this effect was absent in the singular attractor condition (*p* = 0.111, *d* = 0.27). Moreover, there was an effect of attractor number (*p* < 0.001, *d* = 0.68) when comparing the brain responses in the two ungrammatical verbs, with the plural attractor condition eliciting larger negativity than the singular attractor condition. However, the comparison of brain responses in the two grammatical verbs did not show any effect of attractor number (*p* = 0.661, *d* = 0.07).

Moreover, the omnibus ANOVA showed a grammaticality by laterality by an anterior/posterior interaction. However, *post-hoc* comparisons revealed no significant effect of grammaticality in any region (*p*s > 0.1, *ds* < 0.2).

Finally, the omnibus ANOVA also revealed a grammaticality by attractor number by laterality interaction. Follow-up comparisons based on the three-way interaction revealed a significant effect of grammaticality in the plural attractor condition at left, medial, and right sites (*p* = 0.003, *d* = 0.51; *p* = 0.006, *d* = 0.47; and *p* = 0.01, *d* = 0.44, respectively), however, the effect of grammaticality within the singular attractor condition did not reach the significance at left, medial. and right sites (*p*s >0.1, *d*s < 0.2).

#### 500–800 ms Time Window

Statistical analyses revealed a main effect of grammaticality and a significant interaction between grammaticality and anterior/posterior. Follow-up comparisons revealed that ungrammatical sentences elicited significant positivity in the anterior, central, and posterior region (*p* = 0.034, *d* = 0.37; *p* = 0.004, *d* = 0.57; and *p* < 0.001, *d* = 0.70, respectively). Importantly, there was a significant grammaticality by attractor number interaction. Follow-up analyses combining both natives and L2 learners revealed that this interaction was driven by a larger positivity to ungrammatical verbs in the singular attractor condition (*p* < 0.001, *d* = 0.73). However, this effect was absent in the plural attractor condition (*p* = 0.895, *d* = 0.05). Further analyses also revealed a significant main effect of attractor number (*p* < 0.001, *d* = 0.60) when comparing the brain responses in the two ungrammatical verbs, with singular attractors eliciting larger positivity than plural attractors. However, the comparison of brain responses in the two grammatical verbs did not show any differences (*p* = 0.269, *d* = 0.19).

Analyses also revealed a marginal grammaticality by attractor number by group interaction. Due to the interaction with a group, follow-up tests were conducted separately for the two groups. In native speakers, follow-up analyses revealed that the effect of grammaticality was significant in the singular attractor condition (*p* < 0.001, *d* = 0.88), indicating that there is larger positivity evoked by ungrammatical verbs than by grammatical verbs. However, this effect was absent in the plural attractor condition (*p* = 0.704, *d* = 0.08). In L2 learners, follow-up analyses revealed that the effect of grammaticality was only marginally significant in the singular attractor condition (*p* = 0.060, *d* = 0.62), but this effect was absent in the plural attractor condition (*p* = 0.840, *d* = 0.05).

#### Individual Difference Analyses

**Figure 7:**
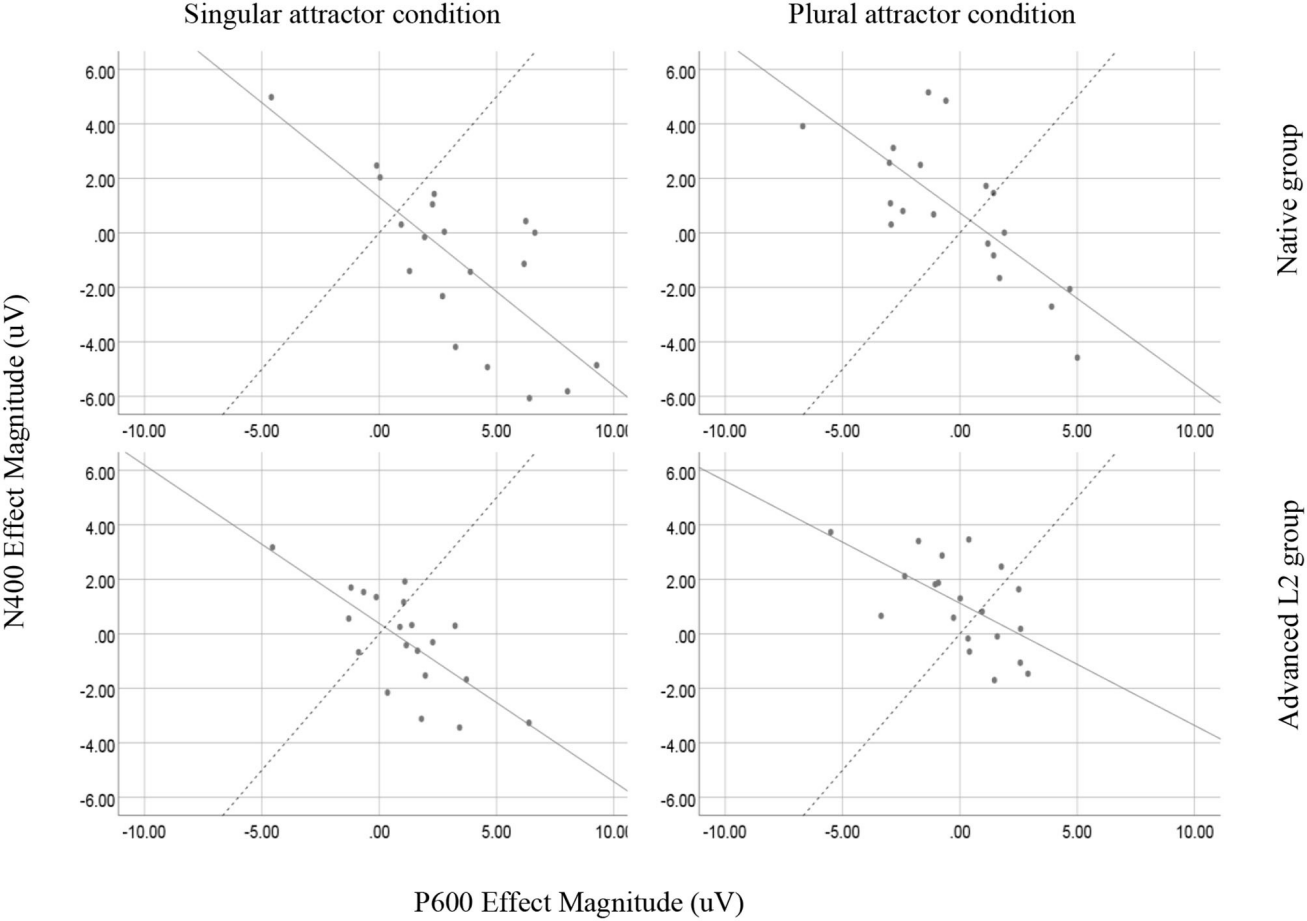
Scatterplots showing the relationship between individuals' N400 effect amplitudes (grammatical minus ungrammatical; y-axis) and P600 effect amplitudes (ungrammatical minus grammatical; x-axis) for both the singular attractor and plural attractor conditions in the two groups, averaged over midline electrodes (Fz, Cz, and Pz). Solid lines show the least squares best fit line; the dashed line indicated the axis of equal N400 and P600 effect amplitudes. Individuals above/to the left of the dashed line of negativity-dominant brain responses; individuals below/to the right of the dashed line have positivity-dominant brain responses.

According to Tanner et al. (2014), we further investigated the brain response profile of individuals. We first computed the magnitude of the brain activity in 300–500 ms by subtracting the mean amplitude of the ungrammatical condition from the mean amplitude of the grammatical condition in both singular and plural attractor sentences, averaged over midline electrodes; the magnitude of the brain activity in 500–800 ms was computed by subtracting the mean amplitude of the grammatical condition from the mean amplitude of the ungrammatical condition in both singular and plural attractor sentences, again averaged over midline electrodes. We then regressed the magnitude of the N400 effects onto that of P600 effects across both conditions in natives and L2 learners. The results showed that the two effects were highly and negatively correlated in both native speakers (singular attractor condition: *r* = −0.721, *p* < 0.001 and plural attractor condition *r* = −0.590, *p* = 0.006) and L2 learners (singular attractor condition: *r* = −0.756, *p* < 0.001 and plural attractor condition *r* = −0.745, *p* < 0.001), as illustrated in Figure 7. This negative correlation revealed an N400-P600 continuum across individuals, with an increase in one effect accompanied by a decrease of the other effect. Lastly, we together pooled the data in both native speakers and L2 learners and tested whether there was still the existence of correlation between the N400 effect and P600 effect in singular and plural attractor conditions. The result again showed that the two effects were negatively correlated (singular attractor condition: *r* = −0.739, *p* < 0.001 and plural attractor condition *r* = −0.688, *p* < 0.001), which suggested a continuity between native speakers and L2 learners in the sensitivity of attraction effects.

## Discussion

This study investigated attraction effects in late advanced Chinese learners of English, focusing on how attractor numbers modulated the grammaticality of agreement processing. According to the results, advanced L2 learners and native speakers exhibited qualitatively similar ERP patterns to attraction manipulation in the processing of verbal agreement: relative to grammatical verbs, a P600 effect was found for ungrammatical verbs in the singular attractor condition, whereas this positivity was absent and instead an N400 effect was found in the plural attractor condition, showing a reliable interference effect of attraction. However, there was still a quantitative difference in agreement processing between the two groups. The P600 effect in L2 processing was less robust than that in native processing, as evidenced by the marginally significant P600 effect with medium effect size (*d* = 0.60) elicited by L2 learners compared with the robustly significant P600 effect with a large effect size (*d* = 0.88) evoked by native speakers. These findings were consistent with the predictions and accounts of SSH in the following aspects. Firstly, the quantitative difference in the P600 amplitude in the singular condition and the sameness in the N400 amplitude in the plural condition between native speakers and L2 learners can be accounted for by SSH, which posits a gradual difference between native and L2 processing, suggesting that adult learners are guided by the shallow parsing route during syntactic comprehension in the same way as native speakers, but less so by the deep parsing route (Clahsen and Felser, 2006b). Secondly, the findings of the P600 effect elicited in the singular attractor condition and the N400 effect in the plural attractor condition in both native and L2 processing can be interpreted as supporting the dual mechanism account of SSH. This account posits that neither native speakers nor L2 learners are restricted to a specific processing route (Clahsen and Felser, 2018), with the possibility of processing syntax by employing the deep parsing route for L2 learners and the shallow parsing route for native speakers. Moreover, the individual difference analyses exhibited that native speakers and L2 learners showed a continuous distribution between the N400 effect and P600 effect, further proving that both groups were able to apply shallow and full parsing routes under even one certain (singular or plural attractor) condition. Lastly, previous studies always took the qualitatively similar pattern of native and L2 syntactic processing as evidence against the validity of SSH. However, in the revised version of SSH, Clahsen and Felser (2018) claimed that L2 learners, as long as they are proficient enough in L2, can be indistinguishable from native speakers in terms of complex syntactic processing. Therefore, the qualitatively similar ERP profile of attraction effects observed in native speakers and L2 learners does not suffice to undermine the theoretical bases of SSH. The major research question posed in the current investigation is whether late advanced L2 learners could show native-like sensitivity to attraction effects. As previously discussed, although L2 learners exhibited qualitatively similar profiles of attraction effects with native speakers, there was a quantitative difference between native and L2 syntactic processing as the amplitude of P600 effect evoked by the L2 group was smaller than that of the native group. These findings indicated that L2 processing of complex agreement with attractor interference was shallower and less automatic than native processing, resulting in difficulty in retrieving and integrating syntactic information in real-time sentence comprehension. For this reason, we suggested that L2 learners cannot exhibit sensitivity to attraction effects in a native-like manner.

Previous self-paced reading and ERP experiments have reported similar sensitivity to attraction effects between native speakers and advanced L2 learners (Tanner et al., 2012; Jegerski, 2016); however, native (Spanish/English) and target languages (English/Spanish) of their participants exhibited the same attraction pattern. Only two studies have investigated attraction effects in native Chinese speakers, whose L1 does not have a systematic number marking system as English does (Jiang, 2004; Chen et al., 2007). Jiang (2004) found no effects of attraction in L2 processing of grammatical sentences. This finding is consistent with the present study, given that we also observed no ERP differences between the two grammatical conditions. However, different from our results, the ERP experiment by Chen et al. (2007) showed that L2 learners exhibited a biphasic N400-P600 pattern when processing agreeing verbs following plural attractors, indicating attraction effects for grammatical verbs. We suggested that the discrepancy may be accounted for by the differences in proficiency levels. In Chen et al. (2007), participants were defined as “proficient” based on College English Test (CET) level four and six that were lower than our selecting standard of TEM level eight. Moreover, their L2 self-proficiency reading rating (4.64) was also lower than that (5.59) of our L2 participants. Owing to their relatively low proficiency level, L2 participants in Chen et al. (2007) may only engage in the shallow processing of local relation between the attractor and verb. When the number features of plural attractors were mismatched with the features of singular agreeing verbs, violations would be detected by participants and thus elicited corresponding ERP results. In contrast, L2 learners in the current study were highly proficient so that they could overcome the local attraction and focus directly on the global agreement between the subject head noun and the verb in grammatical conditions.

The different ERP patterns observed in different attractor conditions in both native and L2 groups were of most interest in our results. In the singular attractor condition, ungrammatical verbs elicited a P600 effect compared to the grammatical verbs. However, in the plural attractor condition, an N400 effect was found at ungrammatical verbs relative to their counterparts. P600 found in the singular attractor condition was predictable because P600 was widely reported to be engendered by syntactic violations like subject-verb agreement anomalies (e.g., Hagoort et al., 1993; Osterhout and Mobley, 1995). However, N400 in the plural attraction condition was somehow unpredicted because this ERP effect was usually regarded as an indicator of semantic violations (e.g., Kutas and Hillyard, 1980). To account for this data pattern, we take our cue from the ERP studies that observed either N400 effect instead of P600 effect or the opposite pattern (P600 effect instead of N400 effect) in thematic role violations (Bornkessel et al., 2002; Kolk et al., 2003; Kim and Osterhout, 2005; Kos et al., 2010). Kos et al. (2010) found that sentences like “*Fred eats in a sandwich…/Fred eats a restaurant…*” elicited an N400 effect rather than a P600 effect. Compared to the baseline conditions such as “*Fred eats in a restaurant…/Fred eats a sandwich…,”* the violation conditions exchanged the phrasal type from PP to NP or from NP to PP. The obvious difference in syntactic cues regarding the phrasal type (NP and PP), as suggested by the authors, is necessary to resolve the conflict at the syntactic level, which leads to a higher processing load at the semantic level and thus eliciting an N400 effect. Some other studies reported the opposite pattern from the results of (Bornkessel et al., 2002; Kuperberg et al., 2003; Kim and Osterhout, 2005; Kos et al., 2010). These studies presented sentences like “*Every morning at breakfast the eggs would eat…*.” While violations of these sentences were seemingly semantic in nature, the combination of content words—for instance morning, breakfast, eggs, and eat (Kuperberg et al., 2003) can form a plausible semantic scenario. As unambiguous lexical-semantic associations can be successfully established, the conflict between the plausible scenarios with syntactic structures, which stipulates that eggs are the agent of the verb “eat,” leads to a higher processing load at the syntactic level, as reflected by the P600 effect. By comparing the study that found a P600 effect instead of an N400 effect to those that observed the opposite pattern in thematic role violations, the difference lies on the relative weights of the syntactic and semantic constraints, with violations carrying more weights of syntactic constraints than the semantic ones eliciting a P600 effect and more semantic constraints than the syntactic ones eliciting an N400 effect. This conflict between the syntax and semantics in sentence processing is well-explained by the dual processing account given by Kuperberg (2007). He proposed two neural processing streams in language comprehension corresponding to the N400 and P600 effect, respectively. The N400 effect reflects a semantic, memory-based system that analyzes the lexical or associative relation between words in sentences; and the P600 effect reflects a syntactically driven combinatorial processing route, which is not only sensitive to morphosyntactic constraints but also interfaces between the lexical-semantic memory and morphosyntax. The account of Kuperberg (2007) seems to be compatible with SSH, which posits two different parsing routes in which the full parsing involves fully specified grammatical presentation base on syntactic information, and shallow parsing provides heuristically driven construction of a “rough-and-ready” representation based on lexical-semantic information and associative pattern (Clahsen and Felser, 2006c). Applying the dual- pathway model of language processing, Tanner (2011) hypothesized that agreement comprehension can proceed along the two parsing streams: the heuristic processing stream, which evaluates lexical associations between agreeing constituents, and the deep processing stream, which establishes the subject-verb agreement relation in the use of morphosyntactic features. This is consistent with the findings observed in this study. The P600 effect found in the singular attractor condition might indicate that subjects took advantage of the full or combinatorial strategy to process the morphosyntactic relationship between the head and verb as the singular attractor could not interfere with agreement computation. However, in the plural attractor condition, subjects were more inclined to make use of syntactically shallow processing to establish dependencies between the verb and the head noun. This change of processing strategy might result from the intervention of the mismatching plural attractors, which “cut off” the syntactic relation between agreeing elements. Assuming that, when plural attractors interfered and caused more difficulties in the comprehension of agreement, it is quite possible that participants might turn to a shallow or “good enough” way to establish the association of lexical forms, rather than agreement features between the head and the verb. Indeed, a similar profile of ERP response has been also found by Barber and Carreiras (2003) who investigated the processing of gender and number agreement in Spanish and observed that disagreement in word pairs elicited the N400 effect, whereas the violation with the same words in sentences engendered a biphasic LAN-P600 pattern. They suggested that the N400 effect reflected the lexical integration of agreeing constituents, and the P600 effect reflected the analysis of syntactic integration of agreement.

It is necessary to note that the results for the native group in our experiment appeared to be in contrast with those reported in previous relevant studies, where attraction effects lead to a smaller amplitude of the P600 effect (Kaan, 2002; Tanner et al., 2014, 2017), the absence of the posterior early negativity (Shen et al., 2013) or frontal negativity (Santesteban et al., 2017), as well as the modulation of different components (Severens et al., 2008). We will first discuss the discrepancies between the present experiment and those in Kaan (2002) and Tanner et al. (2014, 2017). In this study, the individual difference analysis found that native speakers varied along the N400-P600 continuum in both attractor conditions, with the dominance of P600 effects in the singular attractor condition and the dominance of N400 effects in the plural attractor condition. These findings implied that the N400 effects might arise from an increasing number of individuals who showed N400 effects when the attractors were plural. Furthermore, a significant negative correlation between the N400 and P600 effects revealed that those who showed the negativity in the 300–500 ms time window also showed the negativity in the 500–700 ms time windows, and the same was also true for the distribution of the positivity. In the plural attractor condition, it may be the case that the co-occurring negativity in the 500–700 ms time was robust enough to eliminate the positivity in the same time window, such that the positivity was absent in this time window and negativity was circumscribed by the 300–500 ms time window. Extrapolating this pattern of individual difference to the studies by Kaan (2002) and Tanner et al. (2014, 2017), the reduced P600 amplitude found in their experiments might arise from the increasing occurrence of N400 effects. It might be the case that the number of individuals who showed N400 effects increased in the plural attractor condition, but their amplitudes were not strong enough to overwhelm the positivity in the 500–700 ms time window, thus smaller P600 effects were detected. From this perspective, our results seemed to be consistent with those found by Kaan (2002) and Tanner et al. (2014, 2017) in that attraction effects were reflected by a growing bias toward the N400 end along the N400/P600 continuum. It should be emphasized that the above explanation on the difference between our findings and those of Kaan (2002) and Tanner et al. (2014, 2017) is merely a hypothesis, further investigations, especially those with individual difference analyses, need to be done to test its validity. Moreover, although N400/P600 dichotomy in singular and plural conditions was also found by Severens et al. (2008), it showed the opposite pattern from our findings. This difference may originate from the different tasks demanded in experiments. In the present experiment, the grammaticality judgment task required participants to consciously check the agreement violation, which would lead to the elicitation of the P600 effect in the singular attractor condition as no interference took effect. However, in the plural attractor condition, the explicit task might incline participants to use shallow or good-enough strategies to bypass the interference caused by attraction effects, thus eliciting the N400 effects. In contrast, all the questions used in the experiment of Severens et al. (2008) were relevant to the adjective, for example, a question for the sentence “The street near the church/churches is/^*^are beautiful” (English translation form Dutch) could be: “Is the street beautiful?” This type of question was likely to bias the participants toward the semantic reading of sentences, such that they were more focused on the lexical or semantic association rather than morphological features between agreeing elements especially when there was no attraction inference in the singular attractor condition. However, in the plural attractor condition, inconsistencies in the number between the head noun and the attractor noun would force participants to shift their attention from the lexical or semantic processing caused by the experimental question toward the syntactically based combinatorial processing of agreement so as to overcome attraction interferences, thus eliciting the P600 at disagreeing verbs. Finally, it should be admitted that the distinctions between our results and those of Shen et al. (2013) and Santesteban et al. (2017) were harder to explain as more complex factors may be involved. For example, Shen et al. (2013) applied an auditory experimental paradigm and Santesteban et al. (2017) employed an antecedent-clitic dependency structure to investigate attraction effects. Nonetheless, their different findings compared to ours might tentatively prove that the use of different experimental paradigms or syntactic structures may tap into different processing routes with regard to attraction effects.

Importantly, just like native speakers, L2 learners also showed an asymmetrical pattern of attraction effects, in that plural attractors interfered with ungrammaticality at disagreeing verbs, modulating both the N400 and P600 effects, but it did not cause any difficulty in processing grammatical sentences at agreeing verbs. This outcome revealed that there might exist a shared mechanism underlying the general processing of agreement. This could be accounted for by the cue-based working memory retrieval processes (Wagers et al., 2009; Dillon et al., 2013). According to Wagers et al. (2009), a searching process would be initiated to find a possible controller of agreement relationship when encountering a verb. In the case of ungrammatical condition, the plural attractor overlaps in number features with the ungrammatical verb and therefore is likely to replace the singular head noun as the controller of agreement. However, in the condition of grammatical sentences, an agreement dependency can be established between the subject head noun and the verb due to the matching of singular number features of these two items. In this case, the plural attractor will have no more chance to interfere with the already established agreement. To summarize, as both native speakers and L2 learners showed attraction asymmetry, it is conceivable that cue-based working memory retrieval mechanisms could operate in the general processing of subject-verb agreement.

## Conclusion

To conclude, our results showed that late L2 learners, with a high level of proficiency, exhibited qualitatively similar ERP patterns to attraction effects with native speakers: a P600 effect was elicited by ungrammatical verbs when the attractor was singular, and an N400 effect was elicited by incorrect verbs when the attractor was plural. However, the amplitude of the P600 effect found in native processing is quantitatively larger than that in L2 processing, indicating that, compared with native speakers, L2 learners tend to underuse syntactic information or full parsing route during real-time sentence comprehension. These findings were consistent with SSH, which held that the syntactic representations of L2 learners in the comprehension were shallower and less detailed than those of native speakers. Moreover, the individual difference analyses revealed that both L2 learners and native speakers exhibited an N400-P600 continuum in the sensitivity to attraction effects. This outcome was in compliance with the multi-pathway model of SSH, which posits that both native and L2 processing can make use of full and shallow parsing routes. Finally, our results also revealed an asymmetrical pattern of attraction, suggesting the shared working memory retrieval mechanism underlying attraction effects in L2 learners as native English speakers.

As framed in SSH, the results of this study are compatible with this theory in terms of the predication of dual parsing routes for both native speakers and L2 learners, as well as the gradual native/L2 differences in real-time sentence processing. However, there are still some other misunderstandings and misinterpretations of SSH awaiting to be resolved, such as “L2 processing is not subject to L1 influence,” “grammatical processing in L2 learners is qualitatively different from L1, regardless of L2 proficiency or L1–L2 pairing.” For this reason, future studies may take the factors of L2 proficiency, L1 typological distance/proximity into account to clarify SSH in a more comprehensive way. Moreover, the individual difference analyses conducted here only exhibited the distribution patterns of the N400 and P600 effects for both native speakers and L2 learners, but it is still not clear what are the factors that result in such ERP profile patterns. Therefore, future studies can carry out multiple regression analyses (Faretta-Stutenberg and Morgan-Short, 2018) so as to demonstrate how variability in L2 factors, such as motivation, age of acquisition, and working memory, yield quantitative differences in the ERP patterns of attraction effects between native and L2 groups.

## Data Availability Statement

The raw data supporting the conclusions of this article will be made available by the authors, without undue reservation.

## Ethics Statement

The studies involving human participants were reviewed and approved by School of Foreign Languages and Cultures Nanjing Normal University. The patients/participants provided their written informed consent to participate in this study.

## Author Contributions

JB was responsible of data collection and analysis. HZ and CS for the paper writing and guidance of the experiment. All authors made contributions to the design and implementation of the experiment.

## Funding

This article was supported by grant from the National Social Science Fund of China(20AYY010).

## Conflict of Interest

The authors declare that the research was conducted in the absence of any commercial or financial relationships that could be construed as a potential conflict of interest.

## Publisher's Note

All claims expressed in this article are solely those of the authors and do not necessarily represent those of their affiliated organizations, or those of the publisher, the editors and the reviewers. Any product that may be evaluated in this article, or claim that may be made by its manufacturer, is not guaranteed or endorsed by the publisher.
